# Microsatellite loci characterized in the leaf-cutter ant *Atta laevigata*

**DOI:** 10.1186/1756-0500-6-328

**Published:** 2013-08-17

**Authors:** Sérgio Kakazu, Alexandra Sanches, Maurício Bacci Jr

**Affiliations:** 1 Centro de Estudos de Insetos Sociais, Instituto de Biociências, UNESP - Univ Estadual Paulista, Rio Claro, SP 13506-900, Brazil; 2 Departamento de Ecologia, Instituto de Biociências, UNESP - Univ Estadual Paulista, Rio Claro, SP 13506-900, Brazil

**Keywords:** Attini, Molecular systematics, Pest ant, Population genetics, Speciation

## Abstract

**Background:**

The leaf-cutter ant *Atta laevigata* (Formicidae: Attini) is an agricultural pest largely distributed in the Neotropics and a model organism for studies of evolution, speciation and population genetics. Microsatellites are a very powerful tool for these kind of studies, but such markers are not available for studies on *A*. *laevigata*. In the present report, we describe the isolation and characterization of nine microsatellite loci in *A*. *laevigata* and the testing of these markers across other species of leaf-cutter ants.

**Findings:**

Nine microsatellite loci, consisting of six dinucloeotide, one trinucleotide, one tetranucleotide, and one di/trinucleotide repeat motifs, were isolated and characterized. Primers and protocols were successfully designed to selectively amplify these markers. To test effectiveness of these markers for detailed population genetic studies, we genotyped female workers collected from 36 monogynic nests of *A*. *laevigata* and found that eight loci were within Hardy–Weinberg expectations, while the remaining locus had a deficiency of heterozygotes. Micro-Checker analysis of individuals from 55 monogynic nests indicated that loci Alae11, Alae24, Alae18 showed signs of null alleles. For the remaining six loci, the number of alleles per locus ranged between 2 and 11, with expected heterozygosity ranging between 0.07 and 0.88. All of these loci cross-amplified in other species of *Atta*.

**Conclusions:**

These six polymorphic microsatellite loci should prove useful for future genetic investigations of the pest species *Atta laevigata*, as well as studies of other species of leaf-cutter ants in the genus *Atta*.

## Findings

*Atta laevigata* (Smith 1858) belongs to a group of ants called leaf-cutter ants, representing 15 described species in the genus *Atta* and 24 species in the genus *Acromyrmex*[1]. The broad distribution of *A*. *laevigata* throughout the Neotropics [2] makes this species a model organism for detailed phylogeographic studies [3]. Based on variation in morphology, previous authors proposed that *A*. *laevigata* should be divided in two different species [4]. However, morphological differences supporting this division are currently attributed to caste and biogeographic variations within a single species [5]. More recently, two sympatric and distinct morphotypes of this ant have been characterized (Bezerra et al., pers. com). Therefore, it seems that a very recent and perhaps still ongoing process of speciation is taking place in *A*. *laevigata*. Thus, this ant is a promising model organism for studies of speciation. One means of testing for incipient speciation is to evaluate patterns of gene flow between these two sympatric forms.

Microsatellite markers proved to be very effective for estimating gene flow between recently isolated species [6]. Unfortunately, no nuclear microsatellite markers have been described for *A*. *laevigata*. This paper presents a characterization of nine nuclear microsatellite loci derived from *A*. *laevigata*.

In order to isolate microsatellite loci, we constructed an enriched library for tetranucleotide loci following the methodology of Hamilton et al. [7]. Total genomic DNA was extracted from a single specimen of *A*. *laevigata* as per the protocol of Martins et al. [8] and digested with two restriction endonucleases, BstUI and XmnI. DNA fragments (400–1000 bp) were eluted from an agarose gel using the GFX PCR DNA and Gel Band Purification Kit (Ge HealthCare, Buckinghamshire, UK). The enrichment protocol consisted of attaching SuperSNX adapters to fragment ends of eight biotinylated oligonucleotides [(AAAC)_6_, (AAAG)_6_, (AATC)_6_, (AATG)_6_, (ACCT)_6_, (ACAG)_6_, (ACTC)_6_, (ACTG)_6_)] and isolation on streptavidin-coated magnetic beads (Promega, Madison, WI). The recovered DNA was enriched by polymerase chain reaction using the SuperSNX primers, linked into a vector using the CloneJET PCR Cloning Kit (Fermentas, Glen Burnie, MD), and transformed into competent DH10B *E*. *coli* cells.

A total of 384 clones were sequenced in an ABI 3500 automated sequencer (Applied Biosystems, Foster City, CA) using the BigDye Terminator v3.1 Cycle Sequencing Kit (Applied Biosystems, Foster City, CA). Products containing repetitive sequences were identified using the software CID (Clipping Vectors, Identifying SSR, Designing Primers) [9]. Selected sequences were manually inspected for repeats and flanking regions. Primers were designed using Generunner 3.01 (Hastings Software Inc., Hastings on Hudson, NY). In order to characterize and optimize the nine microsatellite loci identified from the initial screening, 36 *A*. *laevigata* female workers in a population in São Paulo state (southwest Brazil) were examined. Each worker was collected from a different monogynic nest.

Polymerase chain reactions were performed using a three primer labeling system [10] in a final volume of 15 μl containing 2 pmol of a specific forward primer, 8 pmol of a specific reverse primer, 8 pmol of M13(−21) universal primer labeled with 6-FAM or NED, 0.125 mM dNTPs, 1× PCR buffer, 1.5 mM MgCl_2_, 1 U Taq DNA Polymerase (Fermentas, Glen Burnie, MD) and ~25 ng template DNA. The amplification protocol was: 95°C (4 min), 35 cycles at 94°C (30 s), 44–56°C (30 s) and 72°C (30 s), followed by 12 cycles at 94°C (30 s), 53°C (45 s), 72°C (45 s) and a final extension at 72°C for 20 min. Amplicons were analyzed with an ABI 3500 (Applied Biosystems, Foster City, CA) automated sequencer and the alleles were scored against the internal GeneScan-600 (LIZ) size standard kit (Applied Biosystems, Foster City, CA).

Tests for Hardy-Weinberg and linkage disequilibrium were performed using Genepop [11,12] and significance values were obtained by the Markov chain method (10,000 dememorization number; 1,000 batches; 10,000 iterations per batch). Bonferroni correction [13] was used to adjust for multiple comparisons and the presence of null alleles for each locus was verified with the Micro-Checker software [14].

For the nine loci examined (Table 1), alleles per locus ranged between 2 and 16 (mean of 8.33), with expected heterozygosity ranging between 0.07 and 0.94 (mean of 0.70). Only Alae18 deviated from the Hardy–Weinberg expectations (Table 1) by displaying a deficit of hetezygotes, which was probably due to the occurrence of null alleles, as detected by the Micro-Checker analysis. This analysis was carried out with a total of 55 monogynic nests (one individual per nest) and, besides Alae18, also indicated that Alae11 and Alae24 are likely null alleles. No pairwise test of the loci for genotypic disequilibrium was significant (*P*-value < 0.0079, following Bonferroni correction).

**Table 1:** **Characterization of nine microsatellite loci isolated from *Atta laevigata***

**Locus**	**Primer sequence (5'-3', forward / reverse)**	**Annealing (°C)**	**Repeat motif**	***N***	***n***_***a***_	**Size (bp)**	***H***_***o***_	***H***_***e***_	***HW p-value***
Alae2	AGTTTCTGCAATATTCGC / TCTGTGAGAGAGCAAGTGAG	49.0	(TC) _1_(TCC) _2_(TC) _9_tt(TC) _4_	25	8	82-98	0.96	0.81	0.4407
Alae5	GCAAAGACATCGTAAAGTG / TGCAACCGTCTTGTATG	52.0	(TAA) _5_	27	2	128-137	0.07	0.07	1.0000
Alae9	TCTTGTAAGTAACTGTCGAGC / CGTCATATCCGAATGTCAG	45.0	(CT)t(CT)_23_	24	11	124-168	0.96	0.88	0.8732
Alae10	CGCTACATCCCATCTCAC / GACAGCAATATTTCGATAGC	44.0	(GA) _19_ca(GA)	27	9	110-130	0.78	0.84	0.0745
Alae11	CACGATAGTTTTCGATATCC / TGGGTGTATCAAAGAAAGAC	45.0	(GA) _15_	26	10	100-128	0.58	0.65	0.3458
Alae16	ACTATGTCCATGTTATGCG / GACTACAAGTAAGAATAGTGAGC	56.0	(TC)_3_tg(TC)_3_tg(TC)_13_tg(TC)_3_tt(TC)_7_	25	7	188 -212	0.72	0.87	0.0145
Alae18	ACATGTCCACTCCGTCAG / CGATAGCGTGATATTTGC	56.0	(TG)_11_	22	16	144-188	0.32	0.94	0.0000*
Alae19	GACGTGGAGCTGCAATAC / AAGTGAGTACAAAACATACAGG	50.0	(CAAA)_5_	22	2	89-93	0.32	0.33	1.0000
Alae24	GCAATAAATTCAGATGGC / CTGCAAAATCACAGTTGC	52.8	(CT)_20_	25	10	169-191	0.72	0.87	0.1904

Number of individuals (*N*), number of alleles (*n*_*a*_), observed (*H*_*o*_) and expected (*H*_*e*_) heterozygosities, p-values of Hardy-Weinberg test (*HW p*-*value*) based on a sample of 36 individuals (* *p*-value < 0.00635, following Bonferroni correction). Given the low amount of DNA from some samples, not all 36 individuals were tested for all primers, although all tested individuals amplified for all loci tested.

Loci Alae2, 5, 9, 10, and 16 were polymorphic for 10 individuals of *Atta robusta*, a species belonging to a different subgenus (Neoatta) from that of *A*. *laevigata* (subgenus Epiatta) [15]. This suggests that the five selected loci should work for most of the *Atta* species occurring in Brazil.

This panel of microsatellite loci will be useful DNA markers for genetic studies of *A*. *laevigata*, providing important data for the understanding of biology, evolution, and genetic diversity of this species. They will be also useful for studies on other leaf-cutter ants in the *Atta* genus.

### Availability of supporting data

The microsatellite sequences are available through the National Center for Biotechnology Information (http://ncbi.nlm.nih.gov) under the accession numbers KC571624 to KC571632.

## Competing interests

The authors declare that they have no competing interests.

## Authors’ contributions

SK and AS searched and characterized microsatellite loci. AS and MB contributed with reagents. SK, AS and MB analyzed the data and wrote the manuscript. All authors read and approved the final manuscript.
